# MRSA outbreak at neonatal ICU in Saudi Arabia

**DOI:** 10.1186/2047-2994-4-S1-P225

**Published:** 2015-06-16

**Authors:** N Dagunton, K Salman, S Al-Saif, B Hanan

**Affiliations:** 1 Infection Prevention & Control Department, King Abdulaziz Medical City Ministry of National Guard, Saudi Arabia; 2 Neonatal Intensive care Unit, King Abdullah Specialized Children’s Hospital, Riyadh, Saudi Arabia

## Introduction

The incidence of MRSA infections at the Neonatal Intensive Care Unit (NICU) of the King Abdulaziz Medical City (KAMC), Riyadh, Saudi Arabia was highest in February of 2014 when an outbreak occurred. Our department conducted a thorough investigation.

The NICU is a 45 bed level 3 unit with an average of 58 per month. Per hospital policy, only neonates delivered at outside facilities are screened nasally for MRSA carriage by the PCR technique

## Objectives

To rapidly identify the cause of the outbreak and implement strict measures to contain the further spread of MRSA.

## Methods

Our investigation employed the following modalities over a nine month period: Placing all neonates in contact isolation as a precautionary measure; conducting weekly Point Prevalence Surveillance screening (PPS); strict enforcement of hand hygiene by health care workers (HCWs) and visiting family members; curtailing of nonessential movement of neonates; enhancement of environmental cleaning protocols with flourescent gel and ATP validation and nasal swab screening of HCWs. Speciation of all MRSA identified was performed by Pulse field gel electrophoresis (PFGE).

## Results

533 and 201 nasal swabs were performed on the neonates and HCWs respectively with a positive MRSA culture in 17 neonates and 5 HCWs. Four different strains of MRSA were identified by PFGE. All 5 MRSA positive HCWs were furloughed and had undergone with MRSA decolonization regimen. Assessment of the environmental cleaning process revealed significant defects.

## Conclusion

Our investigation revealed that the outbreak was not caused by spread of a single MRSA clone. The causes were likely multifactorial and most likely relate to a breakdown of infection control practices, highlighting the importance of strict adherence to infection control practices, including, but not limited to proper environmental cleaning.

## Disclosure of interest

None declared.

